# A Posteriori Reconstruction of the Temperature Distribution in Surface Hardened Tempering Steel

**DOI:** 10.1038/s41598-020-63328-6

**Published:** 2020-05-04

**Authors:** Daniel G. Mevec, Peter Raninger, Petri Prevedel, Vince Jászfi

**Affiliations:** Materials Center Leoben Forschung GmbH, Leoben, 8700 Austria

**Keywords:** Characterization and analytical techniques, Microscopy, Mechanical properties, Metals and alloys

## Abstract

Process control in surface hardening depends greatly on the repeatability of the results. Induction heating facilities stand out in this aspect but challenges arise when it comes to the verification of the expected temperatures. *In-situ* temperature measurement of a workpiece may be made impossible due to it moving through an enclosed, automated induction facility that lacks built-in sensors. This paper uses transition patterns in the microstructure of the hardened region to reconstruct isothermal contour lines of the temperature field during austenitisation. It does so based on a continuous cooling transformation phase diagram and a time-temperature-austenitisation diagram of the considered steel. The presented method serves as a practical approach to validate simulations of the inductive austenitising process and supports simulations of the heat treatment of the work piece. Once these simulations have been iterated upon and validated thoroughly, they may then yield a reconstruction of the entire temperature field during the heat treatment process.

## Introduction

Induction heating techniques have proven a boon to surface hardening, based on their short process times, precise energy input and resulting low energy usage^1^. The repeatability inherent in electronically controlled induction circuits further lends itself to a high degree of automation within a production chain. Process design for induction hardening, however, is non-trivial as the short heating times allow for little diffusion, giving the microstructure of the base material some influence on the properties of the hardened surface^2^. More difficulties are encountered when defining a new geometry, or introducing a workpiece with varying electromagnetic (EM) properties.

In the past, extensive trial and error used up valuable machine time to find new process parameters. Nowadays simulation techniques such as the Finite Element Method (FEM) take on much of the burden by predicting the distribution of heat generation during the heat treatment and inform design decisions before the first test run is scheduled^3–5^. In general, several simplifications and assumptions are always made when simulating a problem (not least of which are estimates of unknowns, such as surface emissivity or heat transfer coefficients) and any simulation needs to be verified in order to produce meaningful data^6,7^.

The most direct way to verify process simulations is to compare the resulting temperature field with data obtained from experiments^7–9^. Temperature data in particular is relatively easy to obtain^10^ – as opposed to the magnetic field distribution within steel parts – and is not contingent on further material models, as the phase or stress distributions are. The concrete difficulty of obtaining the temperature data for a given process can vary wildly from one facility to another. Many modern industrial heat treatment facilities are sealed off from any outside interference, simultaneously increasing the controllability of the process and decreasing the risk of injury due to interaction with moving, conducting, and/or hot parts^11^. These safety and control features come at the expense of accessibility, hindering measurements if no instrumentation has been included during the construction of the facility or the design of the process control software.

Ideally, the heat treatment process is monitored, so that temperature at a certain heating stage or the time dependent temperature of each workpiece is logged, stored, and transferable for quality control and simulations. Often however, this is not the case. On top of that, if the heat treatment process also involves moving workpieces or induction coils, they may obscure line of sight for ad hoc pyrometer measurements and make instrumentation of samples impossible.

The method described in this article deals with such a case, where there was virtually no temperature data available. This was due to the induction facility being highly automated (and therefore enclosed), but not instrumented. While material data could be gained from treated and untreated workpieces, there was no information available on the heat treatment curve the bearing underwent during the process, and recording one was infeasible. The only data point was an estimate of 1050 °C, attained through glimpsing into the induction oven from the intake conveyor and seeing a bright yellow shine through the rotating heating assembly. Needless to say that one temperature value of such questionable origin could hardly be used to verify the rather complex multiphysics simulation that would have to be implemented further in the future.

While there was no way of measuring the temperature *in-situ*, the temperature history of the hardened workpiece still left its traces in its microstructure. This paper aims at describing a method of combining metallurgical data from phase diagrams usually available to the heat treatment facility with a micrograph analysis of a surface hardened sample in order to deduce several depths of different transition temperatures within the material. Consequently, a general numerical temperature distribution fitted to these temperature-depth pairs can be used to verify the estimated surface temperature.

## Experimental Characterization

### Phase diagrams

**Figure 1:**
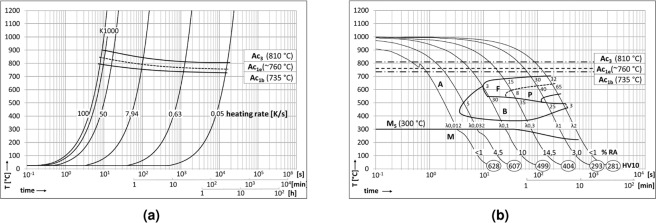
The phase transformation behaviour of the sample steel. (**a**) Displays the time-temperature-austenitisation diagram. Note the shifted transformation temperatures Ac_1_ and Ac_3_ at high heating rates, as well as the split of Ac_1_ into a beginning and an end temperature (Ac_1b_ and Ac_1e_). The heating rate associated with the inductive surface hardening process is marked as K1000 and has a value of 81.67 Ks^−1^. (**b**) Shows the continuous-cooling-transformation diagram evaluated for an austenitisation temperature of 1000 °C with 10 s hold time. A – Austenite, F – Ferrite, P – Pearlite, B – Bainite, M – Martensite, MS – Martensite start, RA – retained austenite, HV10 – Vickers hardness HV10, *λ* – time in seconds from 800 °C to 500 °C divided by 100, 3; 5; 8; … – percentages of final microstructure.

The steel in this study was a modified C38 tempering steel forged into shape and subsequently inductively surface hardened. Its chemical composition is specified by the manufacturer to be according to the ranges listed in Table 1. Its base microstructure was pearlitic-ferritic with a grain size of approximately 30 μm and contained randomly distributed MnS inclusions. These did not affect the hardening process and are irrelevant to the following investigation. The hardening was performed to heat treat a minimum depth of 3 mm from the surface. Samples for dilatometry were cut from untreated zones of the component shafts, measuring 10 mm in length and 4 mm in diameter, and tested using a Bähr DIL805A quenching dilatometer. The phase transformation temperatures were determined at a heating rate (HR) of 3 Kmin^−1^, noting a distinct split of Ac_1_ into a starting temperature Ac_1b_ and an end temperature Ac_1e_. All of these temperatures increase with heating rate, so that the heat treatment in practice, with a heating rate of 81.67 Ks^−1^, experiences Ac_1b_ at 790 °C, Ac_1e_ at 840 °C and Ac_3_ at 895 °C. Differing cooling rates were examined at this heating rate up to an austenitisation temperature of 1000 °C, with 10 seconds of holding time to allow for appropriate austenitisation of the samples. The material exhibits a distinct bainite nose between *λ*0.02 and *λ*0.1. Figure 1 shows the time-temperature-austenitisation (TTA) and continuous-cooling-transformation (CCT) diagrams generated from these experiments.

**Table 1:** Chemical composition as specified by the bearing manufacturer; all data points are given in weight per cent.

C	Mn	Si	P
0.36% to 0.40%	1.30% to 1.45%	0.50% to 0.65%	≤0.025%
**S**	**Cr**	**N**	**Cu**
0.050% to 0.065	0.10% to 0.20%	0.013% to 0.017%	0.25%
**Mo**	**Al**	**Ni**	**V**
≤0.050%	0.010% to 0.030%	≤0.15%	0.08% to 0.12%

### Micrographs

**Figure 2:**
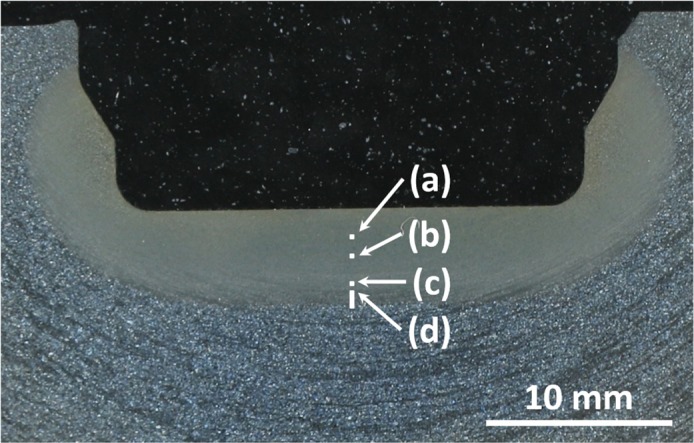
Overview image of the examined microstructure. The marked areas denote the positions at which the micrographs of Fig. 3 were taken. All of the images from Fig. 4 are located at position (d).

**Figure 3:**
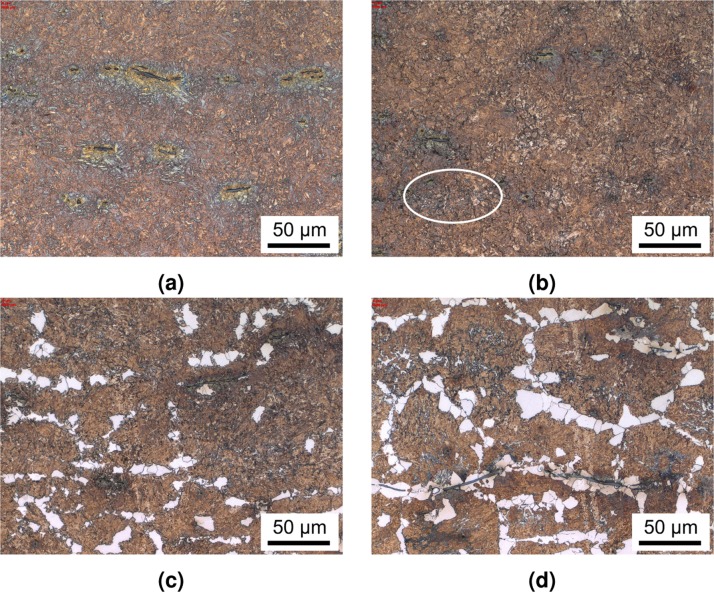
Micrographs of the hardened zone. (**a**) Taken at 1000 μm depth: pure martensite with some manganese sulphides. (**b**) Taken at 2500 μm shows the first occurrence of ferrite (circled) indicating that the material does not entirely reach Ac_3_ any more due to local differences in chemistry (segregations). In (**c**) at a depth of 4000 μm, the original ferritic areas only partly transformed into the austenitic phase, remaining in their original structure of the base metal. In (**d**), the first traces of pearlite are found at 4400 μm, where some of the microstructure did not transform into austenite, indicating that the end temperature for Ac_1_ transformation was not reached at this depth.

**Figure 4:**
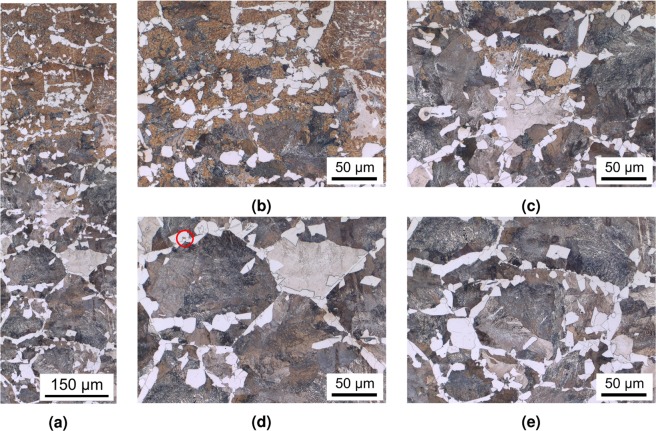
Micrographs of the transition zone: (**a**) shows the entire composite image detailing the changing microstructure in a depth of 4400 μm to 5300 μm from the surface. The first 200 μm are presented in Fig. 3d, with a noticeable overlap of ~20 μm with (**b**), at 4700 μm depth, where untransformed pearlite becomes more pronounced. The amount of martensite is considerably decreasing towards (**c**) with its last traces circled in (**d**), at 5000 μm depth; the vast majority of the microstructure being the original ferritic-pearlitic structure signifies that Ac_1b_ was barely reached. At 5200 μm, shown in (**e**), the microstructure consists entirely of pearlite and ferrite, being consistent with the unaustenitised base metal.

The heat-treated part consists of a bearing journal surface surrounded by flanges. The shaft was cut through the bearing's axis and one journal surface was trimmed to fit into the bedding. The sample was ground and polished with a 1 μm diamond suspension as the finishing step, and subsequently etched using a 3% nitric acid solution, with the prepared sample shown in Fig. 2 as an overview. This and all following micrographs were taken by using a Zeiss Axio Imager M2m optical light microscope with an AxioCam MRc5 installed. The diameter of the bearing is 50 mm while the hardened zone of the material extends to about 5 mm depth at the journal centreline. The surface is austenitised within 12 s and quenched to room temperature within another 10 s, roughly following the *λ*0.012 line in Fig. 1a. The expected microstructure within the hardened zone is therefore purely martensitic. Figure 2 describes the positions of the following micrographs, with Fig. 2(a) through (c) and the upper part of (d) being represented in Fig. 3 and the entirety of (d) shown in Fig. 4.

### Hardness gradient

**Figure 5:**
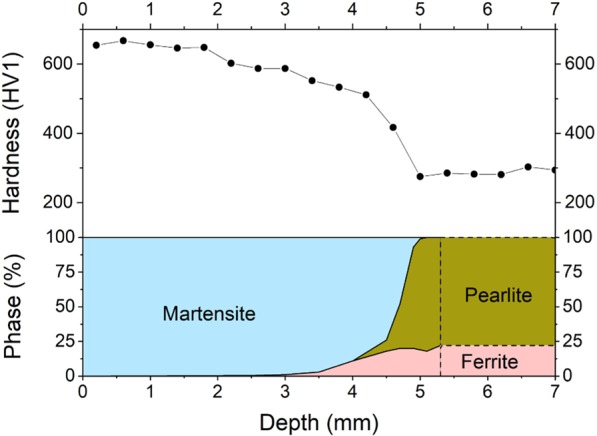
Hardness distribution at the bearing journal centreline showing a plateau of 650 HV1 down to a depth of 2 mm followed by an approximately linear decline to 520 HV1 at 4.2 mm, and a steep drop to a stable hardness of 280 HV1 from 5 mm downward, indicating the original microstructure of the base material. The accompanying phase fractions are shown underneath the hardness, with the microstructure beyond 5.3 mm assumed to be of constant composition.

A line of Vickers hardness measurements was taken along the same centreline as the micrographs, using a Qness Q10A+ Vickers hardness tester. A measuring load of 1 kgf (HV1) was set to allow for close placement of indentations. Figure 5 shows the hardness as a function of depth projected over a phase fraction analysis which was performed on the micrographs shown in Figs. 3 and 4. The hardness gradient is an amalgamation of not only the phase transition, but other influences such as grain size and phase structure. While discerning the exact influences of each effect would go beyond the scope of this report, the hardness is representative of the continuously varying microstructure in the hardened zone. This variation is due to the transformation from ferrite-pearlite to austenite (and later, during quenching, to martensite) by the heat input of the temperature field during the performed inductive surface hardening. Thus the plateau at the end of the hardness gradient corroborates the depth of the Ac_1b_ temperature determined through micrograph analysis.

## Reconstruction of the Temperature Field

The silhouette of the thermal gradient that the material experienced during heat treatment can be guessed from the overview in Fig. 2. However, the detailed analysis in Figs. 3 and 4 reveals the transition zones at which the material crossed the transition temperatures depending on the fast austenitising and short holding time depicted in Fig. 1a. The transformation temperatures Ac_3_, Ac_1e_ and Ac_1b_ are determined at the depths of 2500 μm, 4400 μm and 5000 μm, respectively. The performed hardness measurement corroborates these depths fairly well with average hardness of 650HV1 at the surface coinciding with the predicted 628HV10 of the quenched martensite described in Fig. 1b until a depth of about 2000 μm. While they were attained with a differing load on the Vickers indenter, the resulting hardness values can be assumed to be comparable, as the indentation size effect only starts taking effect at the micro scale (100 gf indentation load)^12^. The subsequent hardness drop can be attributed to the incomplete dissolution of ferrite, which has not completely transformed into the austenitic phase during the heating process, as seen in Figs. 3b–d and 5. An even steeper drop towards the base hardness begins below 4200 μm and corresponds to further increasing amounts of ferrite and the beginning appearance of pearlite (see Figs. 4b,c and 5), indicating the temperature during austenitisation only slightly exceeding Ac_1b_ and thus starting the pearlite to austenite transformation but not completing it.

**Figure 6:**
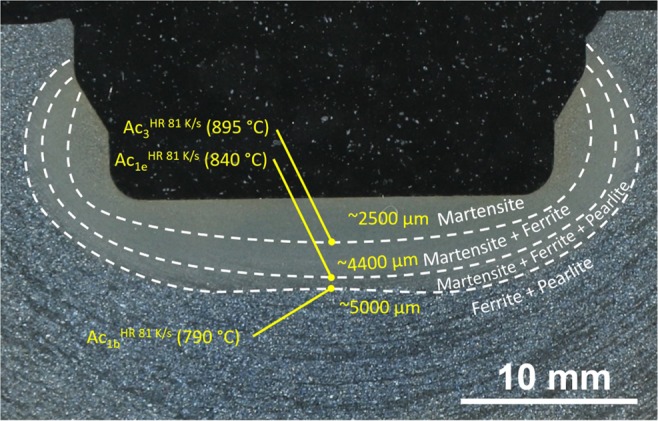
Hardened bearing surface with the three established transition depths at the bearing centre, along with estimated lines of phase transitions and regions of differing microstructures.

The precise depths can be incorporated into the overview image to show an approximation of the transformation zones present during the heat treatment, as depicted in Fig. 6.

The temperature at the surface may still be of interest, since it is the control parameter of choice in most automated induction facilities. Analytical solutions describing the temperature distribution of induction heated cylindrical parts exist^13^ but ignore the cooling of the surface.

**Figure 7:**
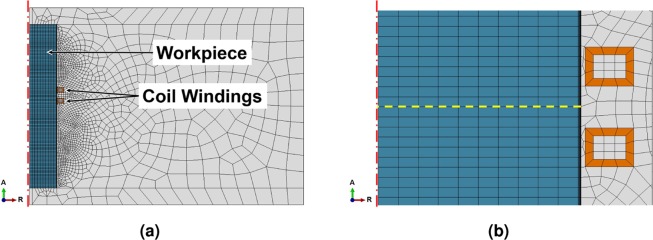
The mesh of the FEM simulation, with the rotational symmetry axis indicated by the red dash-dotted line on the left. The length of the rod above and below the induction coils was chosen to match the mass of the flanges to the sides of the bearing journal, so that it approximates the heat sink of the surrounding material. The yellow dashed line in (**b**) shows the path of the temperature analysis.

A simplified, axisymmetric cylindrical model of the bearing was calculated by FEM simulation with load parameters approximating those of the industrial heat treatment, with detailed parameters given in Table 2 and Fig. 7. The model uses fixed time increments of 250 ms to leapfrog between a linear harmonic solution to the electromagnetic problem and a heat transfer solution that uses heat sources obtained from the previous EM calculation, which provides the temperature distribution for the next EM step. This interaction is regulated by a python script controlling the ABAQUS software used to calculate the results.

**Table 2:** Simulation Parameters for verification.

r^rod^	t^skin^	r_i_^coil^	w^coil^	h^coil^	d^coil^	r^air^
25 mm	349 μm	25.5 mm	6 mm	5 mm	5.4 mm	250 mm
**e**_**0**_^**rod**^	**e**_**0**_^**skin**^	**e**_**1**_^**skin**^	**e**_**∞**_^**air**^	**f**	**I**	**t**_**heat**_
2.5 mm	2 μm	83 μm	15 mm	10.5 kHz	1850 A	12 s

The 5° slice of rod had a radius of r^rod^ = 25 mm and length of l^rod^ = 150 mm. A complex claw-shaped inductor of proprietary geometry encompassed ≈150° of the bearing, which rotated constantly at a distance of 0.5 mm during the hardening process. Since the simplified model was only an axisymmetric slice of the whole circumference, the inductor was represented as a coil of rectangular cross section (w^coil^ = 6 mm wide by h^coil^ = 5 mm tall) with two turns d^coil^ = 5.4 mm apart, distanced 0.5 mm from the bearing surface. The model air space had a radius of r^air^ = 250 mm. Homogeneous Dirichlet boundary conditions were defined at all surfaces; these confined the magnetic field to the simulated geometry by acting as magnetic insulation. This was well suited, since the field was assumed to be axisymmetric and to not extend past the dimensions of the defined cylinder of air. The mesh within the rod was generated from hexagons with a set width of e_0_^rod^ = 2.5 mm. The skin depth was 349 μm and divided into 15 elements geometrically scaled from e_0_^skin^ = 2 μm at the surface to e_1_^skin^ = 83 μm. The air mesh was generated procedurally to scale from 1.5 mm at the coil surface to e_∞_^air^ = 15 mm at the model boundary, while the coils were modelled one element thick with a wall thickness of 1 mm. The load was a sine wave current with an amplitude of I = 1850 A and a frequency of f = 10.5 kHz. This amperage was based on a measurement on the induction coil, but increased slightly to result in a solution close to the assumed maximum surface temperature of 1050 °C. The heat transfer model used only the mesh of the rod, and applied a convective film boundary condition of h_air_ = 20 Wm^−2^K and ambient radiation condition assuming the surface emissivity to be *ε* = 0.7. The initial temperature distribution was set to be a uniform room temperature 25 °C and the rod was heated for t_heat_ = 12 s.

**Figure 8:**
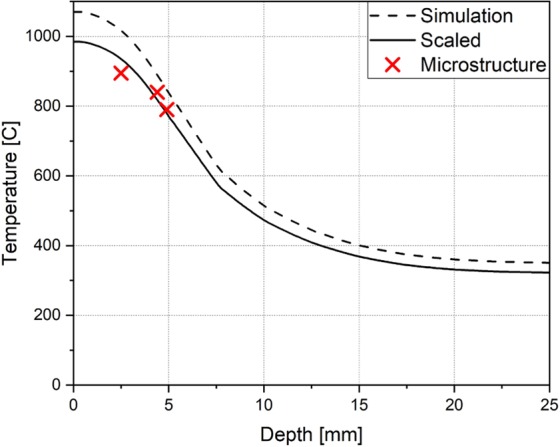
Maximum temperatures reached at each depth as calculated by FEM simulation. The dashed line shows the original calculation reaching a surface temperature of 1071 °C, whereas the solid line has been scaled to minimize the squared differences to the determined transition points in the microstructure. Here the surface temperature reaches 985 °C.

The resulting distribution shows the temperature envelope, i.e. the maximum reached throughout the process, along the path shown in Fig. 7b. While the surface temperature was dialled in to the assumed 1050 °C, it’s envelope was found to be too hight for the observed internal transformation depths. Assuming a linear scaling of the entire distribution, it was fitted to the measured depth of phase transitions and their associated temperatures minimizing the sum of squared residuals. This resulted in an estimated surface temperature of 985 °C (see Fig. 8).

## Discussion

As stated in the introduction, the presented method represents an approach for obtaining temperature data of a surface hardening heat treatment process where there is no *in-situ* measurement possible or available. The thorough investigation of the microstructure in the surface hardened region supplies rather precise ranges of the transition from one microstructural region to another. It is important to note that the transitions are not sharp but rather transitional areas due to local differences in chemistry because of production related segregations and possible variations in the temperature distribution imposed by the inductive heat generation. However, the evaluated transition depths in combination with a TTA-recording for the process conditions supply the transition temperatures for the considered heat treatment quite precisely on a macro scale. The actual transition temperatures in the bearing journal may be somewhat higher, since the TTA-information is drawn from tiny samples with diameter 4 mm that experience homogeneous heating in the dilatometer compared to the 50 mm diameter of the bearing, where a certain degree of overheating is necessary since its core acts as a heat sink during the heating process.

While the transition lines shown in Fig. 6 are based entirely on the evaluation of the microstructure of the central line of the bearing and a qualitative assessment of the overview image, it is of course possible, though work intensive, to generate an arbitrarily fine grid on the etched area, detailing the exact shape of the zones. For verifying a simulation of the heat treatment process, however, a handful of temperatures at different known points is usually sufficient.

The FEM simulation used to extrapolate the surface temperature serves as an example for the verification process: it is a preliminary study using a simplified geometry with estimated process parameters and a linear electromagnetic material model. While the surface temperature fitted in Fig. 8 is close to the expected 1050 °C, the simulation is still in need of calibration and with depth, temperature drops faster than expected. The phase transition regions determined in the microstructure indicate a slower drop of the temperature, which the electromagnetic model needs to be adjusted to account for. Further steps in the modelling procedure now include updating the model geometry, parameters and material model to closer match the physical bearing and result in a better fit with the temperature distribution observed in Fig. 6.

## Conclusions

The following conclusions can be drawn from this work:

- The temperature history at given points on or slightly below the surface of a surface hardened part can be reconstructed based on microstructural features found by a post-mortem microscopy study provided that a time-temperature austenitisation diagram of the material, which has been recorded for process relevant cooling rates, is available.
- FEM simulations of the thermal problem can be validated by comparing the calculated temperature field with those reconstructed temperatures. Unknown simulation parameters such as surface to air heat transfer coefficients can inversely be determined by an iterative approach.
